# Genetic Diversity and Potential Paths of Transmission of *Mycobacterium bovis* in the Amazon: The Discovery of *M. bovis* Lineage Lb1 Circulating in South America

**DOI:** 10.3389/fvets.2021.630989

**Published:** 2021-02-16

**Authors:** Paulo Alex Carneiro, Cristina Kraemer Zimpel, Taynara Nunes Pasquatti, Taiana T. Silva-Pereira, Haruo Takatani, Christian B. D. G. Silva, Robert B. Abramovitch, Ana Marcia Sa Guimaraes, Alberto M. R. Davila, Flabio R. Araujo, John B. Kaneene

**Affiliations:** ^1^Center for Comparative Epidemiology, College of Veterinary Medicine, Michigan State University, East Lansing, MI, United States; ^2^Amazonas State Federal Institute, Manaus, Brazil; ^3^Laboratory of Applied Research in Mycobacteria, Department of Microbiology, Institute of Biomedical Sciences, University of São Paulo, São Paulo, Brazil; ^4^Department of Microbiology and Molecular Genetics, Michigan State University, East Lansing, MI, United States; ^5^Dom Bosco Catholic University, Campo Grande, Brazil; ^6^Agência de Defesa Agropecuaria Do Amazonas, Manaus, Brazil; ^7^Computational and Systems Biology Laboratory, Oswaldo Cruz Institute and Graduate Program in Biodiversity and Health, FIOCRUZ, Rio de Janeiro, Brazil; ^8^Embrapa Gado de Corte, Campo Grande, Brazil

**Keywords:** tuberculosis, bovine TB transmission, clonal complex, whole genome sequencing, spoligotype, *Mycobacterium bovis*, bovine tuberculosis

## Abstract

Bovine tuberculosis (bTB) has yet to be eradicated in Brazil. Herds of cattle and buffalo are important sources of revenue to people living in the banks of the Amazon River basin. A better understanding of *Mycobacterium bovis (M. bovis)* populational structure and transmission dynamics affecting these animals can significantly contribute in efforts to improve their sanitary status. Herein, we sequenced the whole genome of 22 *M. bovis* isolates (15 from buffalo and 7 from cattle) from 10 municipalities in the region of the Lower Amazon River Basin in Brazil and performed phylogenomic analysis and Single Nucleotide Polymorphism (SNP)-based transmission inference to evaluate population structure and transmission networks. Additionally, we compared these genomes to others obtained in unrelated studies in the Marajó Island (*n* = 15) and worldwide (*n* = 128) to understand strain diversity in the Amazon and to infer *M. bovis* lineages. Our results show a higher genomic diversity of *M. bovi*s genomes obtained in the Lower Amazon River region when compared to the Marajó Island, while no significant difference was observed between *M. bovis* genomes obtained from cattle and buffalo (*p* ≥ 0.05). This high genetic diversity is reflected by the weak phylogenetic clustering of *M. bovis* from the Lower Amazon River region based on geographic proximity and in the detection of only two putative transmission clusters in the region. One of these clusters is the first description of inter-species transmission between cattle and buffalo in the Amazon, bringing implications to the bTB control program. Surprisingly, two *M. bovis* lineages were detected in our dataset, namely Lb1 and Lb3, constituting the first description of Lb1 in South America. Most of the strains of this study (13/22) and all 15 strains of the Marajó Island carried no clonal complex marker, suggesting that the recent lineage classification better describe the diversity of *M. bovis* in the Amazon.

## Introduction

*Mycobacterium bovis (M. bovis)* is a member of the *Mycobacterium tuberculosis* complex (MTBC) and is the leading causative agent of bovine tuberculosis (bTB), an OIE (World Organization for Animal Health) notifiable disease that affects mainly cattle, buffalo, and other domesticated and wild animals, but can also be transmitted to humans (zoonotic TB) (1, 2). bTB is distributed worldwide but has very low prevalence in most industrialized countries and has even been eradicated in few nations. However, the disease remains a major problem in developed countries with wildlife reservoirs that end up transmitting the pathogen to domestic livestock and vice-versa, and in developing countries where inefficient bTB control programs result in high disease endemicity and spread (1, 2). In the Brazilian Amazon, few studies aiming to better understand bTB's epidemiology were performed (3–6). The prevalence within animals in the area ranged from 0.1% (4) to 5.4% (6) and the major risk factors associated with bTB were the introduction of new animals into the herds (4), the buffalo species, herds with more than 100 animals, and the presence of cattle and buffalo in the same farm (6).

MTBC members evolved from a most recent common ancestor with *M. canettii*, and are characterized as clonal species demonstrating high genomic similarity (7). Currently, the use of whole-genome sequencing (WGS) to understand tuberculous mycobacteria populational structure is widespread and provided the basis for outbreak tracing and phylogenetic analysis resulting in the classification of human-adapted MTBC into 8 lineages, with *M. tuberculosis* accounting for L1 to L4 and L7-L8, and *Mycobacterium africanum* comprising of L5 and L6 (8, 9). On the other hand, *M. bovis* has been historically classified by Clonal Complexes (CCs), which are identified by genomic deletions, few Single Nucleotide Polymorphism's (SNPs), and/or spoligotypes patterns (10). Accordingly, four different *M. bovis* CCs have been described presenting distinct geographical distribution patterns: African 1 and 2 restricted to Africa, European 2 commonly found in the Iberian Peninsula, and European 1 distributed globally (11–14). With the advent of WGS, recent studies at a global scale provided insights into the population structure and evolution of *M. bovis* lineages (9), showing that CCs do not represent the whole genomic diversity of the isolates (9, 15, 16) and suggesting the existence of at least four *M. bovis* lineages, named Lb1 through Lb4, and three “unknown groups” (9). With the populational structure of *M. bovis* based on WGS starting to be unveiled, additional studies covering different geographic locations are needed to better comprehend worldwide disease spread and to provide new insights regarding the use of genomes to understand disease transmission at the herd and farm levels.

Molecular epidemiological investigation has proved to be a useful tool for TB control and surveillance, which allows us to better understand the dynamics of disease transmission and precisely identify the infectious agent (17). In addition, the knowledge regarding strain diversity within host species has special contribution in areas under risk of zoonotic TB occurrence, thereby providing new insights in strain distribution that may help establishing strategic measures for TB control and prevention (18, 19). In Brazil, few and dispersed molecular epidemiologic studies of *M. bovis* have been reported (20–24), and the WGS characterization of the pathogen is just starting to be unraveled (24–26). While few studies focused on areas of high dairy herd productivity (25, 26), a recent study evaluating *M. bovis* genomes obtained from buffalo and cattle of the Marajó Island, Northern Brazil, was performed and showed the existence of a monophyletic group without CC classification (24). Therefore, the aim of this study was to apply whole-genome and SNP-based phylogenomic analyses to obtain novel information regarding the genetic diversity of *M. bovis* strains circulating in buffalo and cattle from the region of the Lower Amazon River Basin. We believe that such information will guide policy development and strategies to contain the disease in livestock, and thus reduce the risk associated with transmission to humans.

## Materials and Methods

### *M. bovis* Isolate Selection

A total of 24 *M. bovis* isolates were selected, representing one herd of each municipality involved in two previous studies (6, 20) that obtained a total of 63 *M. bovis* isolates from Amazonas State (12 from cattle and 45 from buffalo) and from Pará State (5 from cattle and 1 from buffalo). The selection of *M. bovis* isolates maintained the proportionality according to species (cattle and buffalo) and the local prevalence from the previous studies (6, 20). Accordingly, these isolates were from tissue samples of 8 cattle and 16 buffalos collected at the slaughterhouse from herds with or without known tuberculin skin test (TST) status originating from 12 different municipalities: Alenquer (*n* = 1), Apui (*n* = 1), Autazes (*n* = *1*), Careiro da Varzea (*n* = 1), Itacoatiara (*n* = 2), Manacapuru (*n* = 1), Novo Ceu (*n* = 8), Parintins (*n* = 3), Prainha (*n* = 2), Presidente Figueiredo (*n* = 1), and Urucara (*n* = 3). Samples were collected from June 2016 to October 2017.

### DNA Extraction

*M. bovis* isolates were reactivated in Stonebrink media and incubated until positive growth at 37°C. DNA extractions from colonies suggestive of *M. bovis* for genomic sequencing were performed according to the protocol of van Embden et al. (27), with modifications. Initially, for inactivation, 2-3 colonies were resuspended in 400 μl TE buffer (10 mM Tris-HCI and 1 mM ethylenediaminetetraacetic acid—EDTA, pH 8.0) and heated at 80°C for 30 min. Subsequently, 50 μl of lysozyme (10 mg/ml) were added and incubated at 37°C for 1 h. Then, 75 μl of 10% SDS (sodium dodecyl sulfate) and 10 μl of proteinase K (10 mg/ml) were added and incubated at 65°C for 10 min. Next, 100 μl of 5M NaCl (sodium chloride) and 100 μl of CTAB (cetyltrimethylammonium bromide) were added, followed by stirring and incubation at 65°C for 10 min. After that, 750 μl chloroform/isoamyl alcohol (24:1) were added, stirred and centrifuged at 12,000 g for 5 min. The aqueous phase (surface) was transferred to another tube, 450 μl of isopropanol were added and incubated at −20°C for 30 min and then centrifuged again at 12,000 g for 15 min at room temperature. The supernatant was discarded, and the pellet washed once with 1 ml of ice-cold ethanol (70%) with centrifugation at 12,000 g for 5 min. After drying the tube by evaporation at room temperature, the DNA was resuspended in 20 μl of TE buffer and stored in a freezer at −20°C. The quality and concentration of the extracted DNAs were evaluated using Nanodrop (Thermo Fisher Scientific). Procedures were performed in a Biosafety Level 3 Laboratory located at the Embrapa Gado de Corte, Campo Grande, Brazil.

### Genome Sequencing

WGS was performed at the NGS multi-user platform of Oswaldo Cruz Foundation (FIOCRUZ), Rio de Janeiro, Brazil. Briefly, DNA quantification was performed using the Qubit™ dsDNA HS Assay Kit (Thermo Fisher Scientific, Waltham, USA) and the Agilent High Sensitivity DNA Kit (Agilent, California, USA). WGS of *M. bovis* isolates was carried out on a HiSeq instrument (Illumina, San Diego, CA) using HiSeq Rapid SBS Kit v2 (200 cycles) chemistry and the Nextera DNA Flex Library preparation kit (Illumina, San Diego, CA) according to the manufacturer's instructions. Sequencing reads were deposited in Sequence Read Archive (BioProject number PRJNA675550), NCBI and accession numbers are described in Supplementary Data Sheet 1.

### Genome Quality Assessment and Regions of Difference Identification

Obtained reads were trimmed using a Trimmomatic version 0.38 (28) for adapters and low-quality base removal (sliding window 5:20). Trimmed reads were then evaluated for reads size, per base and read sequence quality, presence of adapters, and GC content using FastQC (29). The GC content had to be around 65% (which is common to mycobacterial genomes) and without multiple peaks (i.e., possible contamination with sequences of different GC content) per quality criteria.

As to confirm that genomes were from *M. bovis*, reads were mapped against *M. tuberculosis* H37Rv using Burrows-Wheeler Aligner (bwa-mem) (30) and the positions according to reference genome of RD1 (4,354,000–4,358,331 nt), RD4 (1,696,017–1,708,748 nt), and RD9 (2,330,880–2,332,100 nt) were evaluated for coverage as previously described (31). The genome was considered as *M. bovis* species when RD1 was absent (i.e. region was intact) and RD4 and RD9 were present (i.e. regions were deleted) (32). Obtained coverage against *M. tuberculosis* H37Rv was also used as quality criteria, considering 95% as a minimum mapping percentage for a genome to be included in the analysis.

### Spoligotyping and Clonal Complexes

Spoligotypes were also investigated *in silico* using SpoTyping (31). Identified genetic spacers were processed in the *M. bovis* Spoligotype Database (www.mbovis.org) to retrieve a spoligotype pattern and SB number. The CCs African 1 (Af1) and 2 (Af2) and European 1 (Eu1) and 2 (Eu2) were evaluated as previously described (9). Briefly, the SNP in the *guaA* gene was investigated in the bam files generated from read mapping against *M. tuberculosis* H37Rv, by checking the position 3,813,236. For the RDs, the same bam files were used to investigate read depth using samtools depth (33) and GNU parallel 2018 (34) for the following regions: RDEu1 (1,768,074–1,768,878 nt), RDAf1 (665,042–668,394 nt), and RDAf2 (680,337–694,429 nt) also as described previously (9).

### Variant Calling

Trimmed *M. bovis* reads were mapped against *M. bovis* AF2122/97 (NC_002945.4) using bwa-mem (30). Duplicated reads were removed using Picard v2.18.23 (https://github.com/broadinstitute/picard). SNPs were called using Samtools v1.9 mpileup (33) and VarScan v2.4.3 mpileup2cns (35), selecting read depth of 7, mapping quality and minimum base quality of 20, and strand bias filter on, followed by annotation using snpEFF (36). INDELs (insertions and deletions), as well as SNPs from repetitive regions (PE/PPE, transposases, integrases, maturase, phage and repetitive family 13E12 genes) were removed from the analysis using a previously described awk command (9). Genomes were also evaluated based on the number of heterogenous SNPs, considering 15% as a maximum amount for a genome to be included in the analysis.

### Phylogenetic Reconstruction

**Figure 1:**
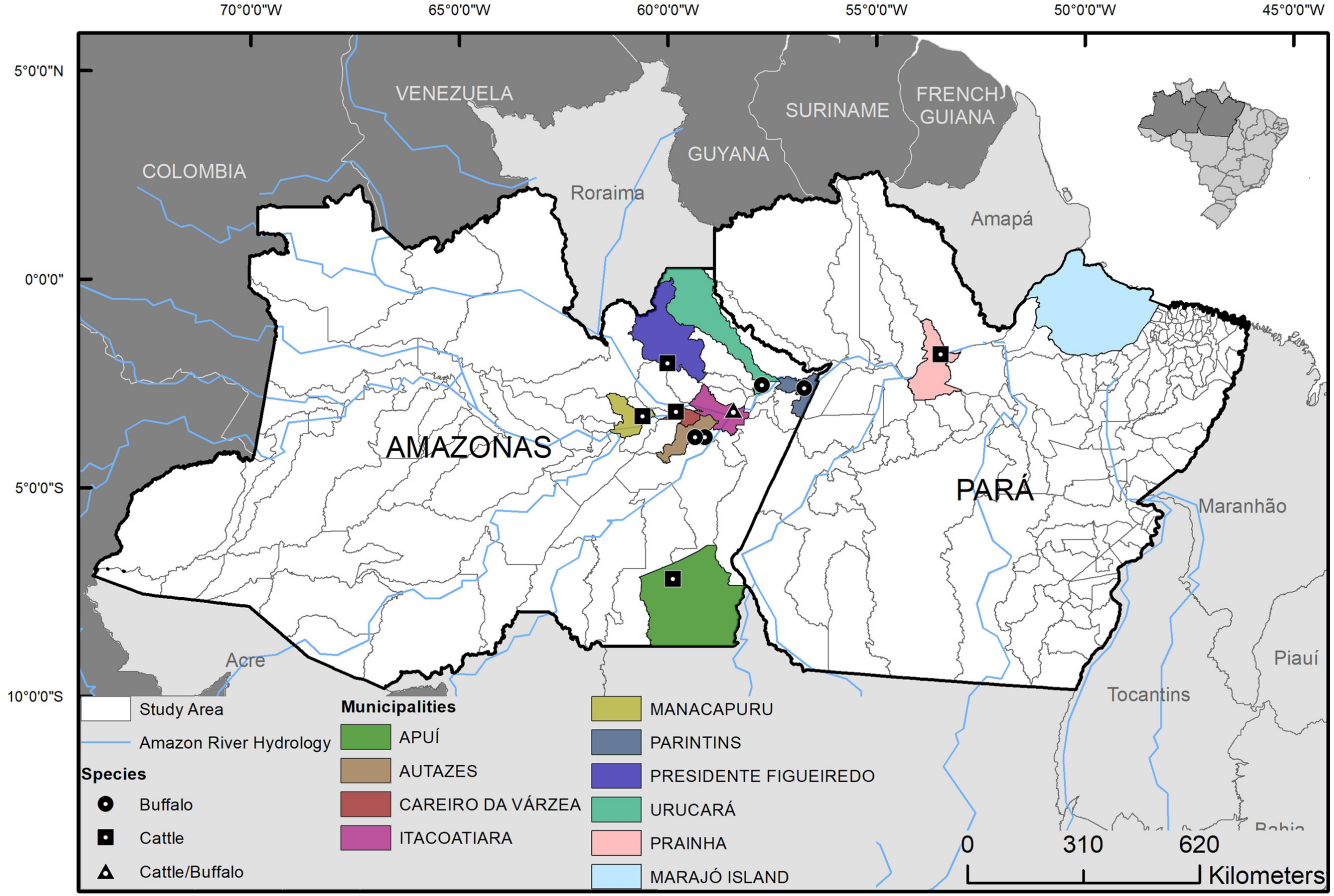
Geographic origin and host species of the *Mycobacterium bovis* isolates from municipalities of Lower Amazon River Basin, Brazil. Isolates were selected from 63 *M. bovis* strains previously isolated from cattle and buffalo (6, 20).

As to evaluate genetic diversity of *M. bovis* in the geographic region, 15 quality-approved *M. bovis* genomes previously sequenced and obtained from cattle and buffalo of the Marajó Island (24) (ENA accession number ERP116404) were used to evaluate the phylogenetic relatedness with the *M. bovis* genomes sequenced in this study. The Marajó Island is geographically close to the targeted region analyzed herein (Figure 1), together with the Lower Amazon River Basin are the regions of major concentration of bufallo in the country, and these *M. bovis* genomes (24) were the only strains of the North of Brazil sequenced up until this study. These genomes were quality-assessed and SNPs were obtained as described above. A matrix of concatenated SNPs of the *M. bovis* genomes was constructed as described (9) and used to estimate a maximum likelihood (ML) phylogeny. RAxML version 8.2.12 (37) was used to construct this phylogenetic tree, selecting the GTRCAT model and autoMRE for best-scoring ML tree and a maximum of 1,000 bootstrap inferences. Genomes of *Mycobacterium caprae* (*M. caprae*) (ERR1462591, ERR1462625, ERR1462617, ERR1462581) were also included in the SNP matrix to serve as outgroup.

### Minimum Spanning Tree

A pairwise SNP-distance matrix and a minimum spanning tree were constructed using PHYLOViZ (38) with default parameters and using a concatenated SNP matrix of the *M. bovis* genomes as input.

### Phylogenomic Analysis With Lineage Representatives of *M. bovis* Genomes

*Mycobacterium bovis* genomes obtained from this study and those from Marajó Island (24) were compared against a collection of 106 genomes representing the four *M. bovis* lineages (Lb1–Lb4) and unknown groups 1, 2, and 3 from a recent study that evaluated worldwide distribution and evolution of *M. bovis* genomes (9) (Supplementary Data Sheet 1). In addition, 21 quality-approved *M. bovis* genomes from France (39), which include representatives of the recently suggested CC called European 3 (40), were also included in the analysis (Supplementary Data Sheet 1). Genomes of *M. caprae* and *M. tuberculosis* H37Rv (outgroup) (Supplementary Data Sheet 1) were included to construct the phylogenetic tree using the ML approach as described above.

### Pairwise SNP-Comparisons

From the SNP-distance matrix, pairwise distances distributions between *M. bovis* genomes obtained from cattle vs. buffalo (Amazon dataset) and between *M. bovis* genomes originating from the Lower Amazon River Basin and Marajó Island were compared using the non-parametric Mann Whitney-test in R software. A result was considered statistically significant when *p*-value ≤ 0.05.

## Results

Out of the 24 *M. bovis* isolates sequenced, two were excluded because of low coverage against the reference genome of *M. tuberculosis* H37Rv or >15% heterogeneous SNPs. These occurred because one isolate resulted in only 38 Kb of sequencing (i.e., failed to be properly sequenced) and the other showed the presence of mixed-strain infection, respectively. The 22 remaining quality-approved *M. bovis* genomes originated from 10 municipalities: Apui (*n* = 1), Autazes (*n* = 1), Careiro da Varzea (*n* = 1), Itacoatiara (*n* = 2), Manacapuru (*n* = 1), Novo Ceu (*n* = 8), Parintins (*n* = 3), Prainha (*n* = 2), Presidente Figueiredo (*n* = 1), and Urucara (*n* = 2) (Figure 1).

The genotypic characterization by spoligotyping revealed 5 distinct profiles (Table 1). The predominant spoligotype was SB0822, detected in 10 isolates, followed by SB0295, representing 6 isolates; the patterns SB1190 and SB1800 were identified in 2 isolates each; the pattern SB0121 had one representative; and one isolate without described spoligotype pattern. Surprisingly, out of 19 *M. bovis* isolates previously typed using an experimental technique (20), 12 were discordant. Disagreement between *in silico* and experimental spoligotyping has been previously described (41, 42). Finally, comparing hosts, our results show a higher number of spoligotypes patterns in buffalo when compared to cattle (Table 1). Given the low sample size, further studies should be conducted to confirm if buffalo have consistently higher diversity of spoligotype patterns when compared to cattle in this region.

**Table 1:** Distribution of *Mycobacterium bovis* in Amazon by municipality, host, and spoligotype.

**Municipality**	**Species**	**Spoligotype**
Apui	Cattle	SB0822
Autazes	Buffalo	Unknown^*^
Careiro da Varzea	Cattle	SB0295
Itacoatiara	Buffalo	SB0295
	Cattle	SB0295
Manacapuru	Cattle	SB0822
Novo Ceu	Buffalo Buffalo Buffalo Buffalo	SB0822 (3) SB1190 (2) SB0295 (2) SB0121
Parintins	Buffalo Buffalo	SB0822 (2) SB1800
Prainha	Cattle Cattle	SB0822 SB0295
Presidente Figueiredo	Cattle	SB0822
Urucara	Buffalo Buffalo	SB1800 SB0822
TOTAL		22

* *Unknown spoligotype pattern: we have submitted the pattern to M.bovis.org database, but up until this publication, a new SB number has not been provided*.

**Figure 2:**
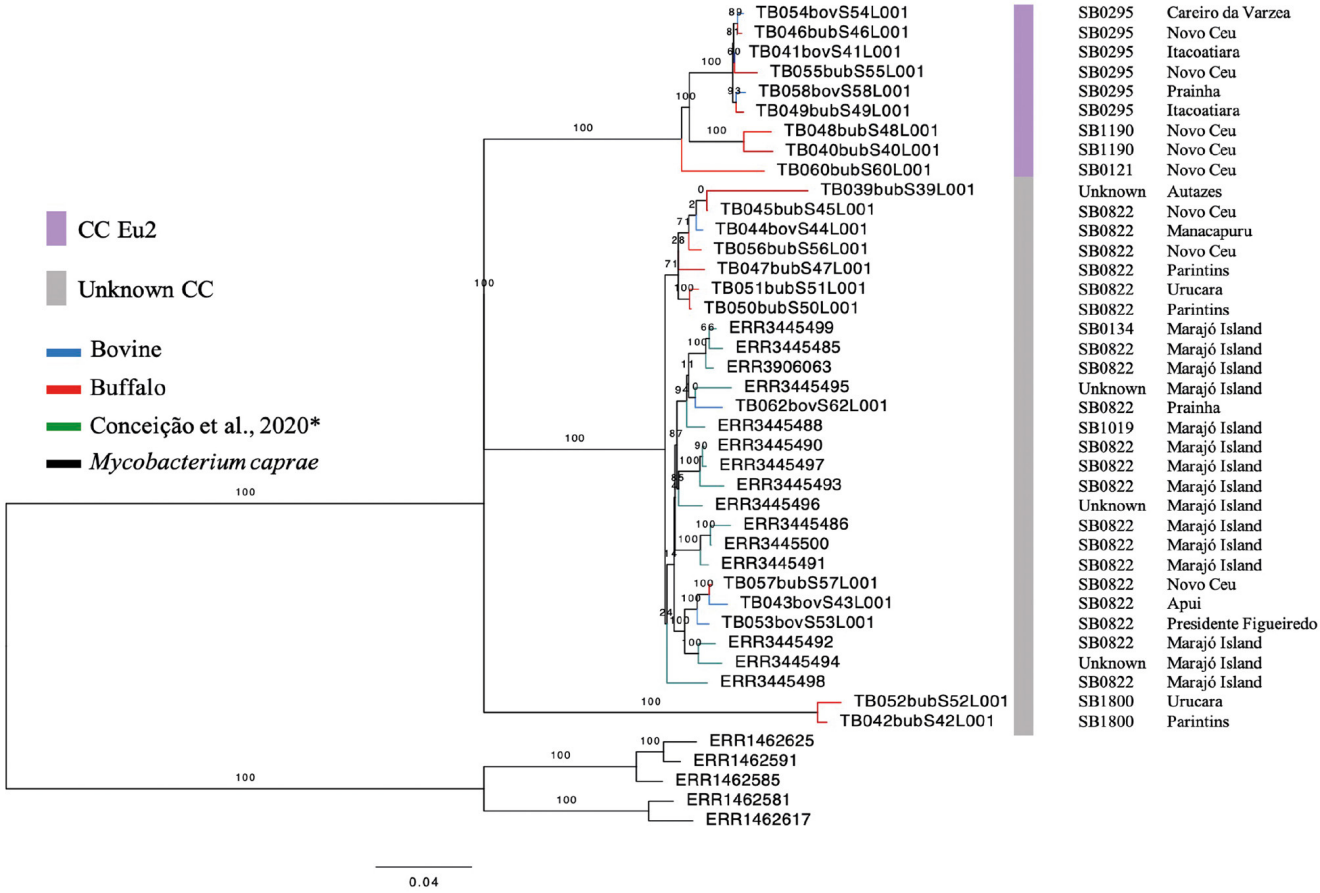
Phylogenetic analysis of *Mycobacterium bovis* genomes from North of Brazil. Maximum likelihood (ML) phylogenetic tree from concatenated SNPs (single nucleotide polymorphisms) of *Mycobacterium bovis* genomes sequenced in this study and by Conceição et al. (24) from Marajó Island. Blue tips: *M. bovis* genomes obtained from cattle and sequenced in this study; red tips: *M. bovis* genomes obtained from buffalo and sequenced in this study; green tips: *M. bovis* genomes from unknown hosts sequenced by Conceição et al. (24) and obtained from cattle or buffalo from Marajó Island, Pará; black tips: *Mycobacterium caprae* genomes (outgroup). Purple bar, *M. bovis* genomes carrying markers of European 2 clonal complex; gray bar, *M. bovis* genomes carrying no markers of known clonal complexes. SB, spoligotype patterns. The phylogenetic tree was generated using RAxML and annotated using FigTree v1.4.3 (43). Horizontal bar shows substitutions per nucleotide. *unknown spoligotype pattern: we have submitted the pattern to M.bovis.org database, but up until this publication, a new SB number has not been provided.

Among the CCs, only the CC Eu2 was found among 41% (9/22) of the isolates. The remaining 13 samples (59%) were not identified as belonging to any known CC (i.e., Eu1, Eu2, Af1, or Af2). Within buffalo, 40% (6/15) of the samples were Eu2, 60% (9/15) had no CC marker. Within cattle, 42.8% (3/7) of the samples demonstrated the marker of CC Eu2, and the remaining of the isolates (4/7) were not classified within any described CC. In the generated phylogenetic tree, with *M. bovis* strains from the Lower Amazon River Basin and Marajó Island, the host classes (buffalo and cattle) are found dispersed among different clades (Figure 2), while *M. bovis* genomes from Marajó Island clustered together, appearing more closely related, and genomes from different Amazonas municipalities did not follow a clear clustering pattern according to geographic region.

**Figure 3:**
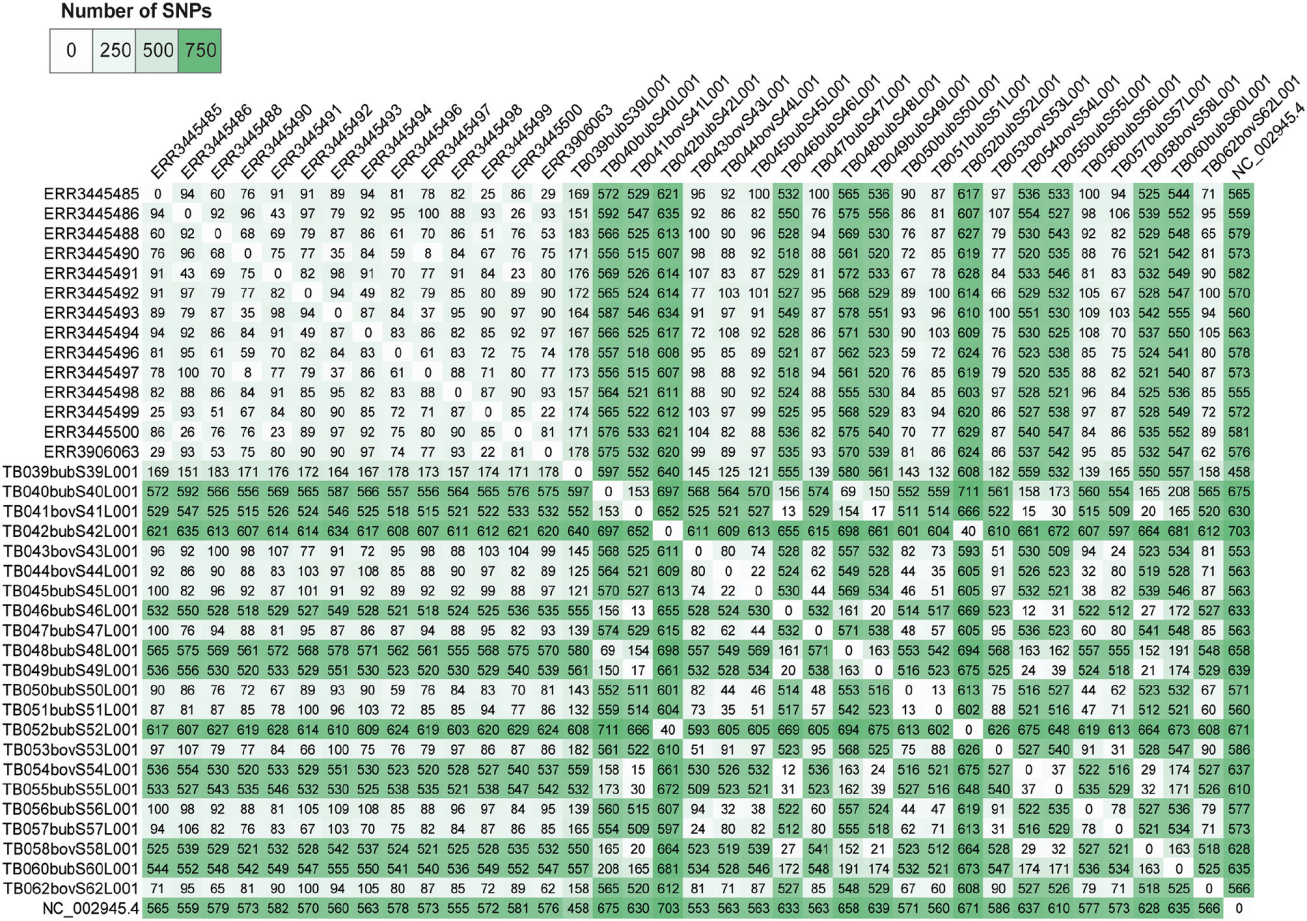
Pairwise single nucleotide polymorphism (SNP)-distance between *Mycobacterium bovis* genomes of Northern Brazil. Genomes of *M. bovis* from the Lower Amazon River Basin (sequenced in this study) and from Marajó Island [sequenced by Conceição et al. (24)] are included. Genomes starting with ERR are from Marajó Island, while genomes starting with TB are from the Lower Amazon River Basin. NC_002945.4: reference genome *Mycobacterium bovis* AF2122/97. Pairwise comparisons were generated from concatenated SNP matrix using PHYLOViZ (38).

**Figure 4:**
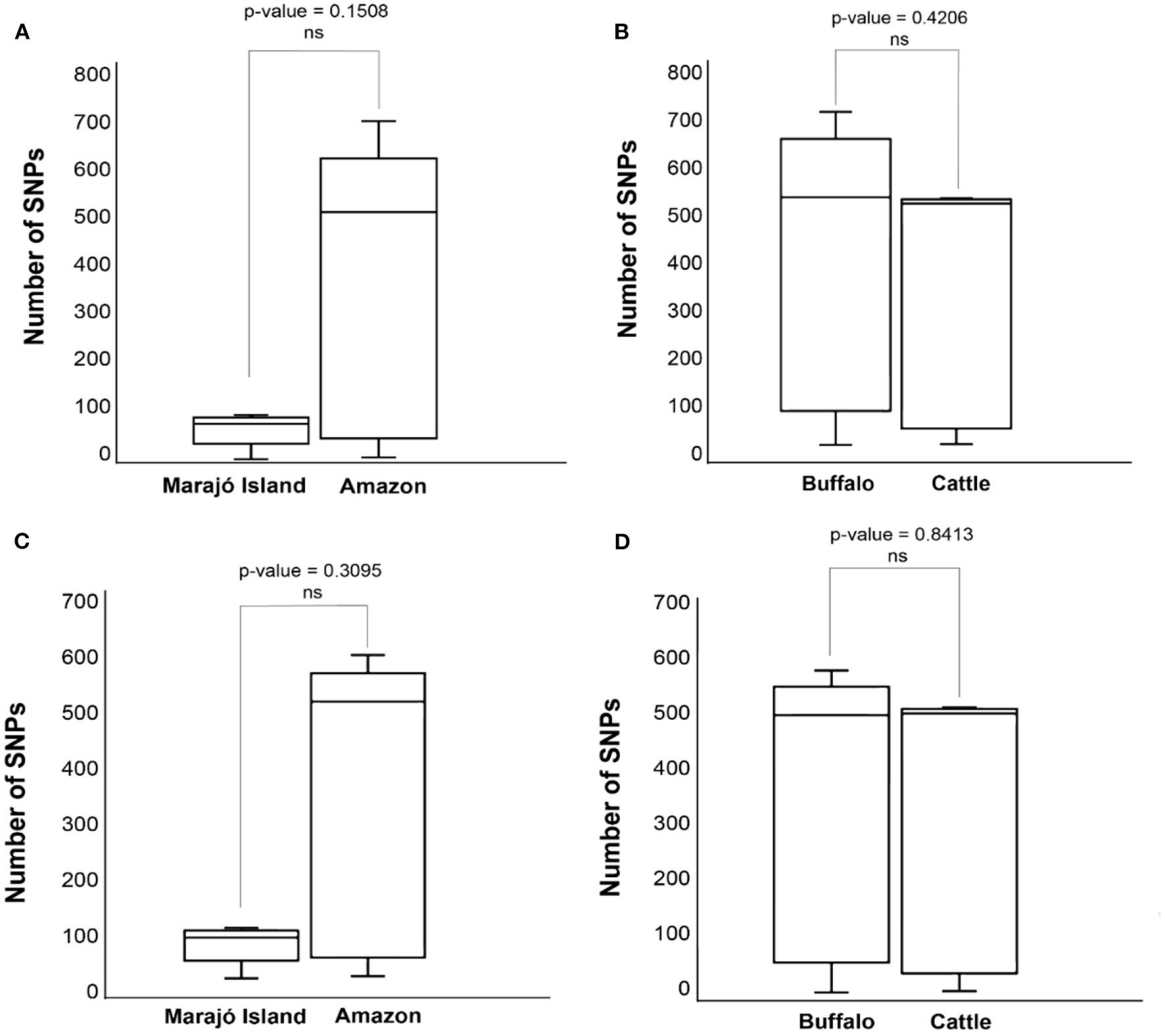
Distributions of pairwise single nucleotide polymorphism (SNP)-distance distance of *Mycobacterium bovis* genomes Northern Brazil. **(A)** Comparison of *M. bovis* pairwise SNP-distance between genomes originating from the Lower Amazon River Basin (sequenced in this study) and from Marajó Island [sequenced by Conceição et al. (24)]. **(B)** Comparison of *M. bovis* pairwise SNP-distance between genomes obtained from buffalo and cattle in the Lower Amazon River Basin. **(C)** Comparison of *M. bovis* lineage 3 (Lb3) pairwise SNP-distance between genomes originating from the Lower Amazon River Basin (sequenced in this study) and from Marajó Island [sequenced by Conceição et al. (24)]. **(D)** Comparison of *M. bovis* Lb3 pairwise SNP-distance between genomes obtained from buffalo and cattle in the Lower Amazon River Basin.

**Figure 5:**
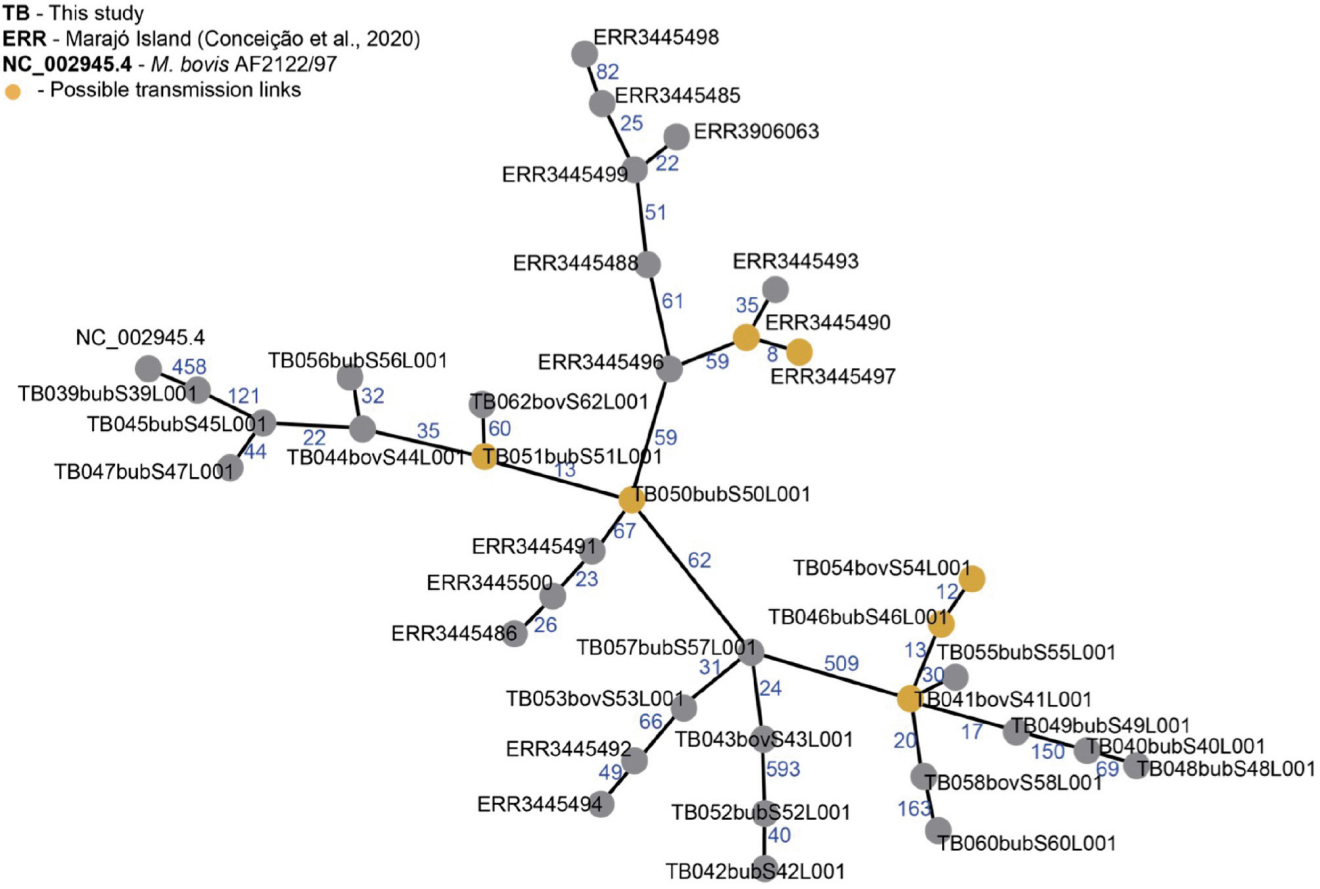
Minimum spanning tree (MST) of *Mycobacterium bovis* genomes from Northern Brazil. Genomes of *M. bovis* from Amazonas state (sequenced in this study, starting with TB) and from Marajó Island [sequenced by Conceição et al. (24), starting with ERR] are included. NC_002945.4: reference genome *Mycobacterium bovis* AF2122/97. Nodes represent *M. bovis* genomes and edges the number of SNPs separating two genomes. Orange nodes represent possible transmission links using an approximate cut-off of 12 single nucleotide polymorphisms (SNP) distance between two *M. bovis* genomes. MST was generated from a concatenated SNP matrix using PHILOViZ (38). Possible transmission links: TB054, TB046 and TB041 are from the municipalities of C. da Varzea, Novo Ceu and Itacoatiara; TB051 and TB050 are from Urucará and Parintins; ERR3445490 and ERR3445497 are from Marajó Island.

Pairwise-SNP comparisons (Figures 3, 4) and minimum spanning tree (Figure 5) of the *M. bovis* genomes obtained herein and from the Marajó Island (24) showed a highly diverse genomic dataset, with pairwise SNP-distances varying from 8 to 711 (from 8 to 100 among *M. bovis* genomes from Marajó Island and from 12 to 711 among *M. bovis* genomes of the Amazon) (Figure 3). Although the overall distribution was not significantly different (Figure 4A), *M. bovis* genomes from the Lower Amazon River region tend to show higher pairwise SNP-distances than *M. bovis* genomes from the Marajó Island (Figure 3). There was also no significant difference of *M. bovis* genetic diversity distribution using the dataset from the Lower Amazon River region as a function of host (Figure 4B; host information from Marajó Island was not available in ENA).

Reflecting this high diversity, based on current *M. tuberculosis*-based SNP threshold to infer recent transmission links (~12 SNPs) (44–46), only three possible active transmission clusters can be observed, one in Marajó Island (host unknown), one connecting the municipalities of Urucará and Parintins (between two buffalo, TB051 and TB050), and one connecting the municipalities of Careiro da Várzea, Novo Céu and Itacoatiara (involving cattle and buffalo, TB54, TB46, and TB41), demonstrating recent transmission between different herds and hosts (Figure 5). Isolates comprising each transmission clusters also had the same spoligotype pattern (SB0822, SB0822, and SB0295, respectively).

**Figure 6:**
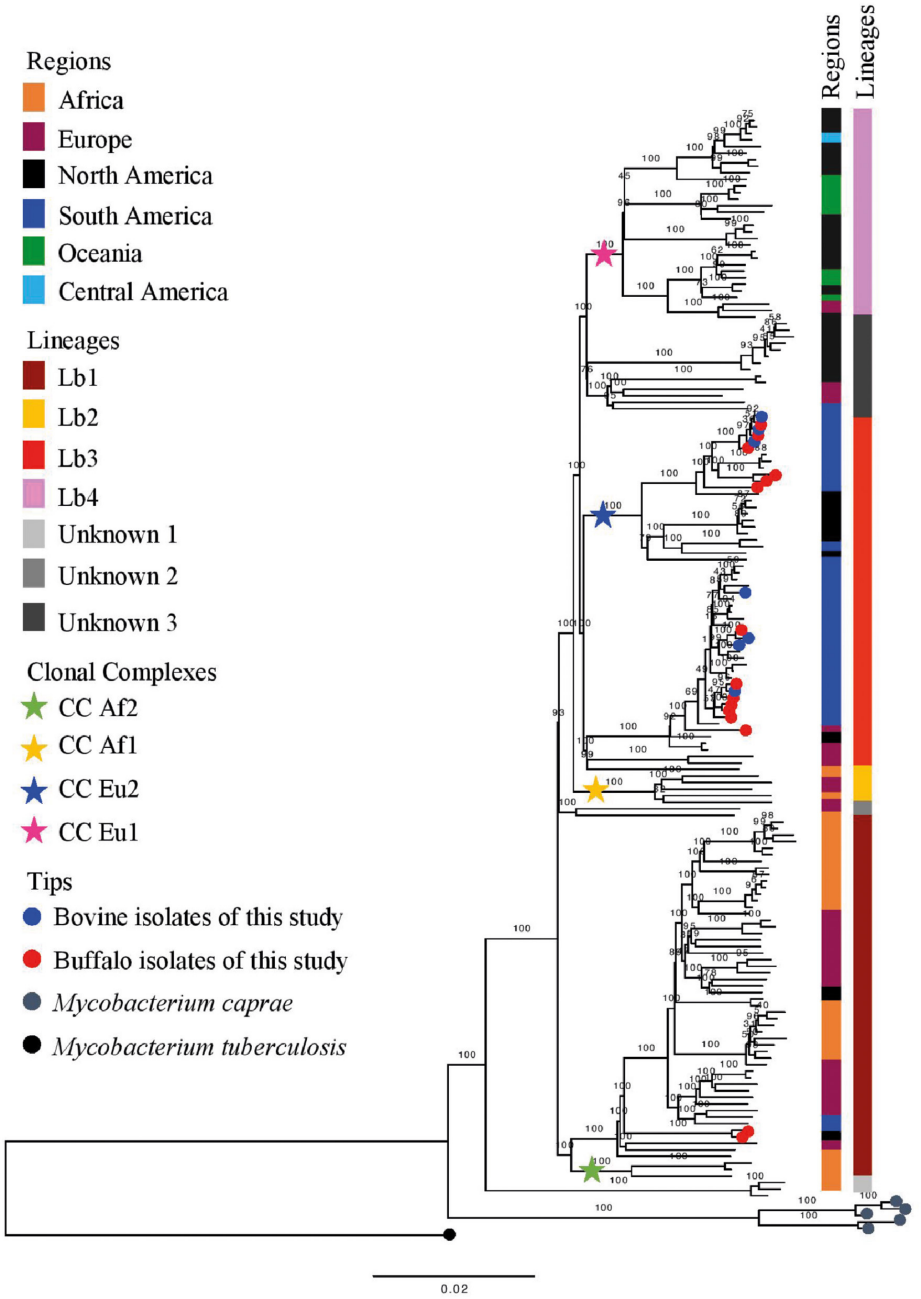
Phylogenetic analysis of *Mycobacterium bovis* lineages. Maximum likelihood (ML) phylogenetic tree from concatenated SNPs (single nucleotide polymorphisms) of *Mycobacterium bovis* genomes from North of Brazil [Amazon, this study, and Marajó Island from Conceição et al. (24)] and worldwide isolates. *Mycobacterium caprae* genomes were included in the analysis, and *Mycobacterium tuberculosis* H37Rv was used as outgroup. The phylogenetic tree was generated using RAxML and annotated using FigTree v1.4.3 (43). Horizontal bar shows substitutions per nucleotide. Lineages are classified according to Zimpel et al. (9).

Based on the proposed four major global lineages of *M. bovis* (Lb1, Lb2, Lb3, and Lb4) (7) our setting would be composed by two lineages, Lb1 with two buffalo isolates (TB052 and TB042) and Lb3 with six buffalo and three cattle isolates with the CC marker Eu2 and seven buffalo and four cattle isolates without CC markers (Figure 6 and Table 2). All isolates from Marajó Island were also identified as being from Lb3 without a CC marker. The presence of Lb1, infecting two buffalo, is the first description of this *M. bovis* lineage in Brazil and requires further investigation into the actual origin of these isolates.

**Table 2:** Distribution of *Mycobacterium. bovis* in Amazon by municipality, year, host, clonal complexes, and lineages.

**Municipality**	**Year**	**Host species**	**Lb1**	**Lb3**	
				**Eu2**	**No CC marker**
Apui	2017	Cattle	-	-	1
Autazes	2017	Buffalo	-	-	1
Careiro da Várzea	2017	Cattle	-	1	-
Itacoatiara	2017	Buffalo	-	1	-
	2017	Cattle	-	1	-
Manacapuru	2017	Cattle	-	-	1
Novo Ceu	2016	Buffalo	-	5	3
Parintins	2017	Buffalo	1		2
Prainha	2017	Cattle	-	1	1
Presidente Figueiredo	2017	Cattle	-	-	1
Urucara	2017	Buffalo	1	-	1
TOTAL			2	9	11

*Eu2, Clonal Complex European 2; CC, Clonal Complex; Lb1 and Lb3, lineages of Mycobacterium bovis 1 and 3*.

In order to evaluate if the high genetic diversity observed in *M. bovis* from the Lower Amazon River region was only due to the presence of the two highly divergent Lb1 genomes, we compared the distributions of pairwise-SNP distances between the Lower Amazon River region and Marajó Island for the Lb3 only (Figure 4C). No significant difference in *M. bovis* genetic diversity was observed (Figure 4C). However, the maximum SNP-distance between two genomes observed in the Marajó Island was 100, while in the *M. bovis* genomes of the Amazon (Lb3 only) continued to be high. There was also no significant difference in the genetic diversity of *M. bovis* Lb3 when comparing genomes obtained from cattle and buffalo (Figure 4D).

## Discussion

To investigate the clonality and population structure of *M. bovis* in the study area at first, we relied on the use of spoligotyping in the characterization of 22 *M. bovis* isolates from 15 buffalo and 7 cattle from June 2016 to October 2017. The spoligotype SB0822 in Brazil was first described in our previous study (20) and we agree with the recent study in Marajó Island in Pará state (24) which seems to demonstrate that SB0822 is the predominant spoligotype in the Amazon region, opposite to a national study with 143 samples from 10 states that found only one occurrence of SB0822 (21). Moreover, SB0822 has been previously described in France (47), cattle in Spain (48) and Portugal (49), and buffalo in Colombia (50), and overall agree with the history of the livestock in the region where the first animals introduced came from Cape Verde (a former Portugal's colony) initially to Marajó Island and from there expanded to the floodplains of the Lower Amazon, on the banks of the Amazon River (51).

Our results also show a higher number of spoligotype patterns of *M. bovis* in the Lower Amazon River Basin compared to the Marajó Island, which can be explained due to the frequent movement of animals in our area of study and the isolation of the herds in the Marajó Island. It is important to highlight that the diversity of *M. bovis* found within buffalo in our sampling is unlikely a result of recent introduction of animals from other Brazilian states since there has been no buffalo imported from other states to the Amazon region and this is probably maintained by constant reinfection from reservoir animals. Accordingly, SB0121 (the most prevalent spoligotyping in Brazil) was found in only one sample, which may reflect the low transit of animals from other states to the area of this study.

Our results show overall higher genetic diversity of *M. bovis* genomes obtained from different municipalities of Lower Amazon River region when compared to the Marajó Island. This is most likely due to the geographic isolation of the island with lower chances of animal importation over time. We also show possible transmission links between buffalo and cattle from different herds but from close geographic proximity. As wildlife reservoirs have not been identified in Brazil thus far, this transmission may have occurred due to infected animal transit and/or introduction into different herds, showing the presence of a two-host system allowing inter-species transmission in the region.

The detection of an inter-species transmission link, the intertwined phylogenetic dispersal of *M. bovis* obtained from cattle and buffalo, and the absence of significant difference in *M. bovis* genetic diversity in cattle *vs*. buffalo suggest that contact rate between different hosts and consequent geographic proximity likely played a more important role in determining the host range of *M. bovis* in this region than host species, agreeing with recent studies (10, 24). Currently the most accepted hypothesis is that *M. bovis* is not a specialized pathogen, i.e., can affect several host species irrespective of its genetic makeup. However, we cannot neglect the possibility of differential host susceptibility to different strains or lineages of *M. bovis*, which may allow one particular strain or lineage to thrive in a specific host. Herein and in the study in Marajó Island (24), for instance, there is only 40% power to determine a possible significant difference in distribution of *M. bovis* strains between buffalo and cattle. Therefore, studies with a higher sample size comparing the dynamics of *M. bovis* infection within buffalo and cattle are needed to definitively clarify this research question.

The results from our study support the fact that current CCs cannot represent the whole diversity of *M. bovis* strains. Interestingly, this is the first time that is described in Brazil isolates of *M. bovis* originating from the *M. bovis* lineage Lb1 (9). In a recent global phylogenomic study of *M. bovis*, strains of Lb1 were shown to emerge from older nodes than Lb3 in the phylogenetic tree and were detected in Eritrea, Ethiopia, Tanzania, Uganda, Tunisia, France, Spain, Italy, Switzerland, and the United States (9). However, some strains identified in this lineage carry the CC Af2 marker (9) which is found at high frequency in bTB cases from East Africa (12), demonstrating the important ties of this lineage to the African continent. One hypothesis to be looked at is that due to the proximity of these countries with Portugal and its colonies; these strains might have been first introduced in the Amazon region during Brazil colonization.

Results from this study, along with others (9, 16, 39, 40), can be used to refine the understanding of *M. bovis* lineage Lb1. With current genomic data, this lineage is composed of two main clusters: one made of *M. bovis* genomes carrying the CC Af2 marker, and the other without known CC markers (9, 16). Herein, the Lb1 cluster without CC Af2 marker includes 19 out of the 21 French *M. bovis* genomes of SB0120 sequenced by Hauer et al. (39) and included in this study. Recently, Branger et al. (52) suggested that this phylogenetic cluster be called CC European 3, also based on their previous work (39). In this study and in others (9, 16), this cluster is composed of *M. bovis* genomes of many spoligotype patterns (e.g., SB0120, SB0134, SB0828, SB0948, SB1517; Supplementary Data Sheet 1) and of BCG vaccine strains (16, 39). Although 19 specific SNPs have been provisionally suggested to be specific of Lb1 (9) and 5 SNPs of its non-Af2 cluster [i.e., CC Eu3; cluster I in Hauer et al. (39)], further studies using comprehensive global datasets should be conducted to confirm or identify definitive genetic markers. The remaining two French *M. bovis* genomes, SRR7851366 and SRR7851376, grouped with genomes of Lb3 and “unknown 3” group, respectively; their disparate position on the phylogenetic tree corroborates the finding of Hauer et al. (39).

The finding of isolates from Marajó Island belonging to Lb3 without CC marker reinforces the previously described (24) existence of a unique *M. bovis* clade in the island that was likely introduced in a single event. In contrast, our results suggest that *M. bovis* was introduced into the Lower Amazon River region as three different events, for which the temporal order remains to be evaluated. One introduction is related to the neighbor cities of Parintins and Urucara, with strains Lb1. Another introduction occurred with Lb3 strains without the CC Eu2 marker, probably with the same origin from the Marajó Island. And finally, an additional introduction is observed with Lb3 strains carrying the CC Eu2 marker, likely from cattle imported from other states and spreading to buffalo. In 2019, a study with 90 samples of cattle lesions suggestive of bTB from the states of Goias, Mato Grosso, Mato Grosso do Sul, Minas Gerais, São Paulo, Tocantins, and Pará found that 14.4% (13/90) belonged to the CC Eu1 and 81.1% (73/90) to the CC Eu2, while 4.65% (4/90) were not identified as any of the four known complexes (53). As the isolates without CC markers were not classified into lineages, the data collected seems insufficient to reveal the true epidemiological picture of the bTB in Brazil. Knowing the genetic profile and understanding the transmission routes of *M. bovis* in the Amazon and elsewhere is essential in order to focus on public health and veterinary resources to contain bTB.

A limitation of this study is the sample size. The small number of *M. bovis* isolates may not be representative of the whole bacterial and animal populations of the Lower Amazon River Basin. Results must be interpreted in light of this fact, and efforts should continue to isolate and study additional *M. bovis* strains from the region.

From our study, we can make the following conclusions: (1) The *M. bovis* CCs classification cannot cover the whole diversity of *M. bovis* strains present in the Amazon region; (2) The presence of *M. bovis* strains Lb1 infecting buffalo requires further investigation into the actual origin of these isolates, showing that the true global diversity of *M. bovis* strains remains to be discovered, likely influenced by cattle trade over history; and (3) The *M. bovis* classification in lineages by SNP-based phylogenetic analyses seems to better cover the diversity of *M. bovis* strains present in the Amazon region compared to CC classification.

## Data Availability Statement

Sequencing reads were deposited in Sequence Read Archive (Bio Project Number PRJNA675550), NCBI and Accession Numbers are described in Supplementary Data Sheet 1.

## Ethics Statement

Ethical review and approval was not required for the animal study because no animal was used in this study.

## Author Contributions

PC and JBK designed experiments, coordinated the research team, interpreted the data, wrote and revised the manuscript. HT and CS coordinated field sampling. CS coordination of field sampling. RA reviewed the manuscript. CZ designed experiments, assessed reads quality, performed phylogenomic analysis, interpreted the data, wrote and revised the manuscript. TTP performed pairwise SNP comparisons, constructed the minimum spanning tree, and interpreted the data. AG designed experiments, supervised CZ and TTP, interpreted the data, wrote and revised the manuscript. FA and TNP cultured *M. bovis*, and performed PCR and DNA extraction. AD performed whole genome sequencing. All authors contributed to the article and approved the submitted version.

## Conflict of Interest

The authors declare that the research was conducted in the absence of any commercial or financial relationships that could be construed as a potential conflict of interest.

## References

[1] Thoen CO Steele JH Gilsdorf MJ. Mycobacterium bovis Infection in Animals and Humans. 2nd ed Ames, IA: Blackwell Publishing (2008).

[2] Chambers MA Gordon SV Olea-Popelka F Barrow PA. Bovine Tuberculosis. Wallingford, Oxfordshire: CABI (2018).

[3] Barbosa JD da Silva JB Rangel CP da Fonseca AH Silva NS Bomjardim HA. Tuberculosis prevalence and risk factors for water buffalo in Pará, Brazil. Trop Anim Health Prod. (2014) 46:513–7. 10.1007/s11250-013-0521-1 24356890

[4] Vendrame FB Amaku M Ferreira F Telles EO Filho JHHG Gonçalves VSP. Epidemiologic characterization of bovine tuberculosis in the State of Rondônia, Brazil. Semin Agrar. (2016) 37:3639–46. 10.5433/1679-0359.2016v37n5Supl2p3639

[5] Silva T Brasil T Moraes R Melo L. First record of bovine tuberculosis in the state of Acre, Brazil. Ciência Anim. (2019) 29:152–7. Available online at: https://www.cabdirect.org/cabdirect/abstract/20203160502

[6] Carneiro PAM Takatani H Pasquatti TN Silva CBDG Norby B Wilkins MJ. Epidemiological study of *Mycobacterium bovis* infection in buffalo and cattle in Amazonas, Brazil. Front Vet Sci. (2019) 6:434. 10.3389/fvets.2019.00434 31921899 PMC6914675

[7] Supply P Marceau M Mangenot S Roche D Rouanet C Khanna V. Genomic analysis of smooth tubercle bacilli provides insights into ancestry and pathoadaptation of *Mycobacterium tuberculosis*. Nat Genet. (2013) 45:172–9. 10.1038/ng.2517 23291586 PMC3856870

[8] Malone KM Gordon SV. *Mycobacterium tuberculosis* complex members adapted to wild and domestic animals. In: Gagneux S, editor. Strain Variation in the Mycobacterium tuberculosis Complex: Its Role in Biology, Epidemiology and Control. Cham: Springer (2017). p. 135–54. 10.1007/978-3-319-64371-7_7 29116633

[9] Zimpel CK Patané JSL Guedes ACP de Souza RF Silva-Pereira TT Camargo NCS. Global distribution and evolution of *Mycobacterium bovis* lineages. Front Microbiol. (2020) 11:843. 10.3389/fmicb.2020.00843 32477295 PMC7232559

[10] Guimaraes AMS Zimpel CK. *Mycobacterium bovis*: from genotyping to genome sequencing. Microorganisms. (2020) 8:667. 10.3390/microorganisms8050667 32375210 PMC7285088

[11] Müller B Hilty M Berg S Carmen Garcia-Pelayo M Dale J Boschiroli ML. African 1, an epidemiologically important clonal complex of *Mycobacterium bovis* dominant in Mali, Nigeria, Cameroon, and Chad. J Bacteriol. (2009) 191:1951. 10.1128/JB.01590-08 19136597 PMC2648362

[12] Berg S Garcia-Pelayo MC Müller B Hailu E Asiimwe B Kremer K. African 2, a clonal complex of *Mycobacterium bovis* epidemiologically important in East Africa. J Bacteriol. (2011) 193:670–8. 10.1128/JB.00750-10 21097608 PMC3021238

[13] Smith NH Berg S Dale J Allen A Rodriguez S Romero B. European 1: a globally important clonal complex of *Mycobacterium bovis*. Infect Genet Evol. (2011) 11:1340–51. 10.1016/j.meegid.2011.04.027 21571099

[14] Rodriguez-Campos S Schürch AC Dale J Lohan AJ Cunha MV Botelho A. European 2—A clonal complex of *Mycobacterium bovis* dominant in the Iberian Peninsula. Infect Genet Evol. (2012) 12:866–72. 10.1016/j.meegid.2011.09.004 21945286

[15] Orloski K Robbe-austerman S Stuber T Hench B Schoenbaum M. Whole genome sequencing of *Mycobacterium bovis* isolated from livestock in the United States, 1989-2018. Front Vet Sci. (2018) 5:253. 10.3389/fvets.2018.00253 30425994 PMC6219248

[16] Loiseau C Menardo F Aseffa A Hailu E Gumi B. An African origin for *Mycobacterium bovis*. Evol Med Public Heal. (2020) 2020:49–59. 10.1093/emph/eoaa005 32211193 PMC7081938

[17] Pérez-Lago L Navarro Y García-de-Viedma D. Current knowledge and pending challenges in zoonosis caused by *Mycobacterium bovis*: a review. Res Vet Sci. (2014) 97:S94–100. 10.1016/j.rvsc.2013.11.008 24360647

[18] Smith NH Dale J Inwald J Palmer S Gordon SV Hewinson RG. The population structure of *Mycobacterium bovis* in Great Britain: clonal expansion. Proc Natl Acad Sci USA. (2003) 100:15271–5. 10.1073/pnas.2036554100 14657373 PMC299979

[19] Drewe JA Smith NH. Molecular epidemiology of Mycobacterium bovis. In: Thoen CO, Steele JH, Kaneene JB, editors. Zoonotic Tuberculosis: Mycobacterium bovis and Other Pathogenic Mycobacteria, third edition. Ames, IW: Wiley Blackwell (2014). p. 79–88.

[20] Carneiro PAM Pasquatti TN Takatani H Zumárraga MJ Marfil MJ Barnard C. Molecular characterization of *Mycobacterium bovis* infection in cattle and buffalo in Amazon Region, Brazil. Vet Med Sci. (2020) 6:133–41. 10.1002/vms3.203 31571406 PMC7036311

[21] Figueiredo Rocha VC de Souza-Filho AF Ikuta CY Hildebrand e Grisi Filho JH de Azevedo Issa M Coelho Mota PMP. High discrimination of *Mycobacterium bovis* isolates in Brazilian herds by spoligotyping. Prev Vet Med. (2020) 179:104976. 10.1016/j.prevetmed.2020.104976 32361639

[22] Zimpel CK Brandão PE de Souza Filho AF de Souza RF Ikuta CY Ferreira Neto JS. Complete genome sequencing of *Mycobacterium bovis* SP38 and comparative genomics of *Mycobacterium bovis* and *M. tuberculosis* strains. Front Microbiol. (2017) 8:2389. 10.3389/fmicb.2017.02389 29259589 PMC5723337

[23] Carvalho RCT Vasconcellos SEG Issa MDA Filho PMS Mota PMPC De Araújo FR. Molecular typing of *Mycobacterium bovis* from cattle reared in midwest Brazil. PLoS ONE. (2016) 11:1–16. 10.1371/journal.pone.0162459 27631383 PMC5024986

[24] Conceição ML Conceição EC Furlaneto IP Silva SP Guimarães AES Gomes P. Phylogenomic perspective on a unique *Mycobacterium bovis* clade dominating bovine tuberculosis infections among cattle and buffalos in Northern Brazil. Sci Rep. (2020) 10:1747. 10.1038/s41598-020-58398-5 32019968 PMC7000724

[25] Anzai EK. Sequenciamento do genoma completo do Mycobacterium bovis como instrumento de sistema de vigilância no Estado de Santa Catarina. São Paulo: University of São Paulo (2019).

[26] Patané JSL Martins J Castelão AB Nishibe C Montera L Bigi F. Patterns and processes of *Mycobacterium bovis* evolution revealed by phylogenomic analyses. Genome Biol Evol. (2017) 9:521–35. 10.1093/gbe/evx022 28201585 PMC5381553

[27] van Embden JD Cave MD Crawford JT Dale JW Eisenach KD Gicquel B. Strain identification of *Mycobacterium tuberculosis* by DNA fingerprinting: recommendations for a standardized methodology. J Clin Microbiol. (1993) 31:406–9. 10.1128/JCM.31.2.406-409.1993 8381814 PMC262774

[28] Bolger AM Lohse M Usadel B. Trimmomatic: a flexible trimmer for illumina sequence data. Bioinformatics. (2014) 30:2114–20. 10.1093/bioinformatics/btu170 24695404 PMC4103590

[29] Andrews S. FastQC: A Quality Control Tool for High Throughput Sequence Data [Online] (2010). Available online at: http://www.bioinformatics.babraham.ac.uk/projects/fastqc/ (accessed January 23, 2021).

[30] Li H Durbin R. Fast and accurate long-read alignment with Burrows-Wheeler transform. Bioinformatics. (2010) 26:589–95. 10.1093/bioinformatics/btp698 20080505 PMC2828108

[31] Faksri K Xia E Tan JH Teo YY Ong RTH. *In silico* region of difference (RD) analysis of *Mycobacterium tuberculosis* complex from sequence reads using RD-analyzer. BMC Genomics. (2016) 17:1–10. 10.1186/s12864-016-3213-1 27806686 PMC5093977

[32] Warren RM Pittius NCG Barnard M Hesseling A Engelke E Kock M. Differentiation of *Mycobacterium tuberculosis* complex by PCR amplification of genomic regions of difference. Int J Tuberc Lung Dis. (2006) 10:818–22. 16850559

[33] Li H. A statistical framework for SNP calling, mutation discovery, association mapping and population genetical parameter estimation from sequencing data. Bioinformatics. (2011) 27:2987–93. 10.1093/bioinformatics/btr509 21903627 PMC3198575

[34] Tange O. GNU Parallel. (2018). Available online at: https://www.gnu.org/software/parallel (accessed January 23, 2021).

[35] Koboldt DC Zhang Q Larson DE Shen D McLellan MD Lin L. VarScan 2: somatic mutation and copy number alteration discovery in cancer by exome sequencing. Genome Res. (2012) 22:568–76. 10.1101/gr.129684.111 22300766 PMC3290792

[36] Cingolani P Platts A Wang LL Coon M Nguyen T Wang L. A program for annotating and predicting the effects of single nucleotide polymorphisms, SnpEff. Fly (Austin). (2012) 6:80–92. 10.4161/fly.19695 22728672 PMC3679285

[37] Stamatakis A. RAxML version 8: a tool for phylogenetic analysis and post-analysis of large phylogenies. Bioinformatics. (2014) 30:1312–3. 10.1093/bioinformatics/btu033 24451623 PMC3998144

[38] Francisco AP Vaz C Monteiro PT Melo-Cristino J Ramirez M Carriço JA. PHYLOViZ: phylogenetic inference and data visualization for sequence based typing methods. BMC Bioinformatics. (2012) 13:87. 10.1186/1471-2105-13-87 22568821 PMC3403920

[39] Hauer A Michelet L Cochard T Branger M Nunez J Boschiroli M-L. Accurate phylogenetic relationships among *Mycobacterium bovis* strains circulating in France based on whole genome sequencing and single nucleotide polymorphism analysis. Front Microbiol. (2019) 10:955. 10.3389/fmicb.2019.00955 31130937 PMC6509552

[40] Branger M Loux V Cochard T Boschiroli ML Biet F Michelet L. The complete genome sequence of *Mycobacterium bovis* Mb3601, a SB0120 spoligotype strain representative of a new clonal group. Infect Genet Evol. (2020) 82:104309. 10.1016/j.meegid.2020.104309 32240800

[41] Xia E Teo Y-Y Ong RT-H. SpoTyping: fast and accurate *in silico* Mycobacterium spoligotyping from sequence reads. Genome Med. (2016) 8:19. 10.1186/s13073-016-0270-7 26883915 PMC4756441

[42] Coll F Mallard K Preston MD Bentley S Parkhill J McNerney R. SpolPred: rapid and accurate prediction of Mycobacterium tuberculosis spoligotypes from short genomic sequences. Bioinformatics. (2012) 28:2991–3. 10.1093/bioinformatics/bts544 23014632 PMC3496340

[43] Rambaut A. FigTree v1.4.3 (2012). Available online at: http://tree.bio.ed.ac.uk/software (accessed January 4, 2021).

[44] Walker TM Ip CLC Harrell RH Evans JT Kapatai G Dedicoat MJ. Whole-genome sequencing to delineate *Mycobacterium tuberculosis* outbreaks: a retrospective observational study. Lancet Infect Dis. (2013) 13:137–46. 10.1016/S1473-3099(12)70277-3 23158499 PMC3556524

[45] Hatherell HA Colijn C Stagg HR Jackson C Winter JR Abubakar I. Interpreting whole genome sequencing for investigating tuberculosis transmission: a systematic review. BMC Med. (2016) 14:1–13. 10.1186/s12916-016-0566-x 27005433 PMC4804562

[46] Merker M Kohl TA Niemann S Supply P. The evolution of strain typing in the *Mycobacterium tuberculosis* complex. Adv Exp Med Biol. (2017) 1019:79–93. 10.1007/978-3-319-64371-7_3 29116629

[47] The Mycobacterium bovis Spoligotype Database. Available online at: http://www.Mbovis.org (accessed January 4, 2021).

[48] Romero B Aranaz A Sandoval Á Álvarez J de Juan L Bezos J. Persistence and molecular evolution of *Mycobacterium bovis* population from cattle and wildlife in Doñana National Park revealed by genotype variation. Vet Microbiol. (2008) 132:87–95. 10.1016/j.vetmic.2008.04.032 18539410

[49] Matos F Cunha MV Canto A Albuquerque T Amado A Botelho A. Snapshot of *Mycobacterium bovis* and *Mycobacterium caprae* infections in livestock in an area with a low incidence of bovine tuberculosis. J Clin Microbiol. (2010) 48:4337–9. 10.1128/JCM.01762-10 20844227 PMC3020884

[50] Jojoa-Jojoa J Maira Wintaco M Francisco Osorio R Puerto-Castro G Guerrero-Guerrero M. First approach to molecular epidemiology of bovine tuberculosis in Colombia. Rev. MVZ Cordoba. (2016) 21:5222–36. 10.21897/rmvz.32

[51] Lourenço Júnior JB Garcia AR. Produção Animal No Bioma Amazônico : Atualidades E Perspectivas. In: An. Simpósios da 43a Reun. Anu. da SBZ Joao Pessoa, PB (2006). p. 42–60.

[52] Branger M Hauer A Michelet L Karoui C Cochard T De Cruz K. Draft genome sequence of *Mycobacterium bovis* strain D-10-02315 isolated from wild boar. Genome Announc. (2016) 4:e01268–16. 10.1128/genomeA.01268-16 27834714 PMC5105107

[53] Sales ÉB de Alencar AP Hodon MA Soares Filho PM de Souza-Filho AF. Identification of clonal complexes of *Mycobacterium bovis* in Brazil. Arch Microbiol. (2019) 201:1047–51. 10.1007/s00203-019-01674-4 31111186

